# Quantitative Proteomics Comparison of Total Expressed Proteomes of *Anisakis simplex* Sensu Stricto, *A. pegreffii*, and Their Hybrid Genotype

**DOI:** 10.3390/genes11080913

**Published:** 2020-08-10

**Authors:** Susana C. Arcos, Lee Robertson, Sergio Ciordia, Isabel Sánchez-Alonso, Mercedes Careche, Noelia Carballeda-Sanguiao, Miguel Gonzalez-Muñoz, Alfonso Navas

**Affiliations:** 1 Department of Biodiversity and Evolutionary Biology, Museo Nacional de Ciencias 8 Naturales, CSIC, calle José Gutiérrez Abascal 2, 28006 Madrid, Spain; scobacho@mncn.csic.es (S.C.A.); robertson.lee@inia.es (L.R.); 2 Departamento de Protección Vegetal, INIA. Ctra La Coruña Km 7’5, 28040 Madrid, Spain; 3 Unidad de Proteómica Centro Nacional de Biotecnología, CSIC, calle Darwin 3, Campus 11 de Cantoblanco, 28049 Madrid, Spain; sciordia@cnb.csic.es; 4 Instituto de Ciencia y Tecnología de Alimentos y Nutrición, CSIC. Calle José Antonio 13 Novais, 10, 28040 Madrid, Spain; isabel.sanchez@csic.es (I.S.-A.); mcareche@ictan.csic.es (M.C.); noeliacarba@hotmail.com (N.C.-S.); 5 Servicio de Immunología, Hospital Universitario La Paz. Paseo de la Castellana, 261, 28046 Madrid, Spain; mgonzalez_munoz@hotmail.com

**Keywords:** *Anisakis simplex* s.s, *A. pegreffii*, quantitative proteomics, iTRAQ, proteins, biomarkers

## Abstract

The total proteomes of *Anisakis simplex* s.s., *A. pegreffii* and their hybrid genotype have been compared by quantitative proteomics (iTRAQ approach), which considers the level of expressed proteins. Comparison was made by means of two independent experiments considering four biological replicates of *A. simplex* and two each for *A. pegreffii* and hybrid between both species. A total of 1811 and 1976 proteins have been respectively identified in the experiments using public databases. One hundred ninety-six proteins were found significantly differentially expressed, and their relationships with the nematodes’ biological replicates were estimated by a multidimensional statistical approach. Results of pairwise Log_2_ ratio comparisons among them were statistically treated and supported in order to convert them into discrete character states. Principal component analysis (PCA) confirms the validity of the method. This comparison selected thirty seven proteins as discriminant taxonomic biomarkers among *A. simplex*, *A. pegreffii* and their hybrid genotype; 19 of these biomarkers, encoded by ten loci, are specific allergens of *Anisakis* (Ani s7, Ani s8, Ani s12, and Ani s14) and other (Ancylostoma secreted) is a common nematodes venom allergen. The rest of the markers comprise four unknown or non-characterized proteins; five different proteins (leucine) related to innate immunity, four proteolytic proteins (metalloendopeptidases), a lipase, a mitochondrial translocase protein, a neurotransmitter, a thyroxine transporter, and a structural collagen protein. The proposed methodology (proteomics and statistical) solidly characterize a set of proteins that are susceptible to take advantage of the new targeted proteomics.

## 1. Introduction

The genus *Anisakis* is formed by 10 known and at least two undescribed species (*Anisakis* sp. 1 and *Anisakis* sp. 2) based on L3 genotypes [1,2]. *A. simplex* sensu stricto, *A. pegreffii*, and *A. berlandi* constitute the *Anisakis simplex* sensu lato (s.l.) complex [3,4,5]. Although the classical taxonomy of *Anisakis* is based on differences found in the excretory system, esophageal intestinal region of larvae L3 [6,7], and the morphology of adult males [8,9], the use of the nuclear ribosomal internal transcribed spacer (ITS) has provided a consistent molecular diagnostic tool for larvae L3 for routine diagnosis [10,11] and also for the detection of recombinant genotypes (hybrids) between *A. simplex* s.s. and *A. pegreffii* [12,13,14]. Both species are the main responsible agents of anisakiasis [15,16,17,18,19] and can co-infect the same fish host [20]. Although no fertile adult hybrids have been found, it is considered that recombinant genotypes are products of interspecific hybridization between *A. simplex* s.s. and *A. pegreffii* [1,21]. These hybrids also express allergenic and immunoreactive proteins with potential clinical or pathogenic implications [22]. Recently tissue specific transcriptomes of *A. simplex* s.s and *A. pegreffii* [23] have been published and compared with *Hysterothylacium aduncum* [24]. In another study, the total transcriptomes of both species and their hybrids [25] in the same development stage (L3) from four common fish hosts (*Scomber japonicus*, *Engraulis encrasicolus*, *Sardina pilchardus*, and *Merluccius merluccius*) were published. The data they provided are basic to compare the metabolic and gene ontology profiles and expression patterns of the three taxonomic entities. In addition, these transcriptomic studies provided resources for studies on host-parasite relationships and the involved pathogenic mechanisms. All of this information can be combined with the final expressed proteome as the final functional system, ultimate responsible for functionality and taxonomic characteristics. Statistical comparison of proteomes is an efficient selection method of biomarkers in what is termed in proteomics “discovery phase” [26,27] Methodological developments in proteomics are applied to research in different topics relating to parasites [28,29,30,31]; however, few are devoted to taxonomic discrimination at a species or population level. The taxonomy of *Anisakis* is very stable and a wide range of intraspecific or genetic differences have been demonstrated [10] for which proteomic analysis can be applied for diagnostic purposes [32]. Limited results have been known on expressed proteome differences regarding any species of this important parasitic nematode genus. Because comparison of protein expression profiles can be treated as quantitative inheritance characters [33], its codification in discrete states would facilitate the finding of unambiguous proteins markers with taxonomic, evolutionary and phylogenetic value [29,34,35]. Here, we applied these assumptions as a proteomic discovery phase [27] to three *Anisakis* taxonomic entities (*A. simplex* s.s., *A. pegreffii*, and their hybrid genotype) in order to characterize and select a unique set of specific proteins as taxonomic markers able to be experimentally targeted (qualification, verification, validation) and also to provide a methodological framework focusing on taxonomical protein markers of other species of the genus *Anisakis* for clinical and food diagnostic purposes.

## 2. Materials and Methods

### 2.1. Taxonomic Identification of Nematodes and Selection of Specimens for Proteins Extraction

Nematodes were collected during a general survey carried out in the Mercamadrid Central Fish Market, related to the incidence of these nematodes in commercial fish species. All specimens were captured in the FAO 27 major fishing area. A total of 235 pools of L3 larvae specimens (12–50) were randomly obtained from eight different fish hosts. Parasites were removed following published procedures [21,36,37]. The larvae pools were rinsed in 0.9% saline solution and placed in an antibiotic-antimitotic solution (80 mg gentamycin sulphate, 0.625 mg amphotericin B, 10.000 IU penicillin G, 10 mg streptomycin sulphate, 4.5 mL of saline Hank’s solution making up to 10 mL of volume with bi-distilled water; Sigma Aldrich, St. Louis, MO, USA). After 40 min in disinfectant solution, the larvae pools were rinsed in bi-distilled water for 1 h. From each larvae pools (from each fish host), all specimen were isolated and for each of these L3 larvae specimens, the caudal part was used for DNA extraction and PCR amplification for species and hybrid identification; this caudal part and the rest of the body (used for proteomics experiment) were separately stored at −80 °C until required. *Anisakis* identification was performed following the taxonomic criteria of D’Amelio et al., 2000 [11] and Abollo et al., 2003 [14] using the ITS1 region of the nuclear ribosomal DNA (rDNA).

#### Selection of Specimens for Proteomics

We selected completely independent biological replicates of nematodes for the proteomics experiments. For *A. simplex* s.s. and *A. pegreffi* pure “specimen populations” were independently selected from six *Merluccius merluccius* hosts. In the case of *A. simplex*, four pools of larvae each containing 10 individuals were prepared as 4 biological replicates (*A. simplex*-1, *A. simplex*-2, *A. simplex*-3, and *A. simplex*-4). For *A. pegreffii* two independent biological replicates each with 10 individuals were prepared (*A. pegreffii*-1, *A. pegreffii*-2). However mixed hybrids genotype specimens were selected from different hosts (*Micromesistius poutassou*, *Merluccius merluccius*, *Conger conger*, *Lepidorhombus boscii*, *Lophius budegassa*, *Lophius piscatorius*, *Scomber scombrum*, *Thunnus thynnus*) in which one or both species and their hybrids were present because no pure hybrid specimen populations were found. The hybrids can differ depending on the contribution of the maternal and paternal genomes (*A. simplex* s.s. or *A. pegreffii*). We have used voucher material previously selected based on the results of mitochondrial DNA sequences, which differentiate the two types of hybrids [26] (data in DB Anisakis, “COIImarkers,” www.anisakis.mncn.csic.es and https://www.ncbi.nlm.nih.gov/bioproject/PRJNA316941) considering each as an independent biological replicate (Hybrid-1 and Hybrid-2) based on the phylogenetic position (resemblance to *A. simplex* or *A. pegreffii*). In total, a mix of six different maternal hybrid specimens were used—for Hybrid-1, six specimen whose COII clusters to *A. simplex* (Vouchers 48.50, 82.19, 92.26, 99.39, 178.6, 187.31) and for Hybrid-2, six specimen whose COII clusters to *A. pegreffii* (Vouchers 45.9, 90.5, 115.18, 119.36, 122.4, 176.22)—guaranteeing the independence of both hybrids biological replicates.

### 2.2. Extraction of Nematodes Proteins

Biological replicates were crushed with a pestle in a 1.5 mL. Eppendorf tube and the extract were suspended in 200 µL of lysis buffer (7M urea, 2M thiourea, 2% (v/v) triton-100, IPG buffer pH 3–11 2% (v/v), 40 mM DTT with protease and phosphatase inhibitors). Total proteins were precipitated using methanol/chloroform [38]. Protein pellets were resuspended and denatured in 7 M Urea/2 M Thiourea/100 mM TEAB, pH 7.5. The final protein concentration was estimated using the RC DC Protein Assay kit (Bio-Rad, Hercules, CA, USA).

### 2.3. Protein Digestion and Tagging with iTRAQ-4-plex^®^ Reagent

For digestion, 40 ug of protein from each condition/population was reduced with 2 µL of 50mM Tris(2-carboxyethyl) phosphine (TCEP, SCIEX), pH 8.0, at 37 °C for 60 min and followed by 2 µL of 200mM cysteine-blocking reagent (Pierce MMTS, methyl methanethiosulfonate; Thermo Fisher Scientific, Waltham, MA, USA) for 10 min at room temperature. Samples were diluted up to 1 M Urea concentration with 25 mM TEAB. Digestions were initiated by adding sequence grade-modified trypsin (Sigma-Aldrich, St. Louis, MO, USA) to each sample in a ratio of 1:20 (w/w), which were then incubated at 37 °C overnight on a shaker. Sample digestions were evaporated to dryness.

Digested samples were labelled at room temperature for 2 h with iTRAQ Reagent Multi-plex kit (SCIEX, Foster City, CA, USA) according to the manufacturer’s instructions. The iTRAQ labelling was performed separately in parallel in two four-plex designs using tags 114, 115, 116, and 117 (Supplementary File 1, Labelling Scheme). To reinforce the criteria of independent comparison, in both labelling (iTRAQ2 and iTRAQ3), tags were used for *A. simplex*-1, *A. pegreffii*-1, *A. simplex*-2, and Hybrid-1 in the case of iTRAQ2, while for iTRAQ3 tags were used for *A. simplex*-3, *A. pegreffii*-2, *A. simplex*-4, and Hybrid-2. After labelling, the samples were pooled, dried and desalted using a SEP-PAK C18 Cartridge (Waters). Finally, the cleaned tryptic peptides were evaporated to dryness and stored at −20 °C until analysis.

### 2.4. Liquid Chromatography and Mass Spectrometer Analysis

Three technical replicates (a 1 µg aliquot of labelled mixture per experiment) were subjected to 1D-nano LC ESI-MSMS analysis using a nano liquid chromatography system (Eksigent Technologies nanoLC Ultra 1D plus, SCIEX, Foster City, CA, USA) coupled to high speed Triple TOF 5600 mass spectrometer (SCIEX, Foster City, CA, USA) with a Nanospray III source. The analytical column was a silica-based reversed phase Acquity UPLC M-Class Peptide BEH C18 Column, 75 µm × 150 mm, 1.7 µm particle size, and 130 Å pore size (Waters). The trap column was a C18 Acclaim PepMap™ 100 (Thermo Fisher Scientific, Waltham, MA, USA), 100 µm × 2 cm, 5 µm particle diameter, 100 Å pore size, switched on-line with the analytical column. The loading pump delivered a solution of 0.1% formic acid in water at 2 µL/min. The nano-pump provided a flowrate of 250 nL/min and was operated under gradient elution conditions. Peptides were separated using a 250 min gradient ranging from 2% to 90% mobile phase B (mobile phase A: 2% acetonitrile, 0.1% formic acid; mobile phase B: 100% acetonitrile, 0.1% formic acid). The peptides were monitored throughout the chromatography and the gradient has the following steps: 0–180 min: 2–30% of B, 180–200 min: 30–60% of B, 200–215 min: 60–90% of B, 215–225 min: 90% of B, 225–230 min: 90–5% of B and 230–250 min: 5% of B. Injection volume was 5 µL.

Data acquisition was performed with a TripleTOF 5600 System (SCIEX, Foster City, CA, USA) using an ionspray voltage floating (ISVF) 2300 V, curtain gas (CUR) 35 L/h, interface heater temperature (IHT) 150 °C, ion source gas 1 (GS1) 25 L/h, declustering potential (DP) 150 V. All data was acquired using information-dependent acquisition (IDA) mode with Analyst TF 1.7 software (SCIEX, Foster City, CA, USA). For IDA parameters, 0.25 s MS survey scan in the mass range of 350–1250 Da were followed by 30 MS/MS scans of 150 ms in the mass range of 100–1800. Switching criteria were set to ions greater than mass to charge ratio (m/z) 350 and smaller than m/z 1250 with charge state of 2–5 and an abundance threshold of more than 90 counts (cps). Former target ions were excluded for 20 s. IDA rolling collision energy (CE) parameters script was used for automatically controlling the CE. The mass spectrometry proteomics data have been deposited to the ProteomeXchange Consortium via the PRIDE [39] partner repository with the dataset identifier PXD019289 and 10.6019/PXD019289.

### 2.5. Data Analysis

The mass spectrometry data obtained for pooled samples were processed using PeakView^®^ 1.5.1 Software (SCIEX, Foster City, CA, USA) and exported as mgf files which were searched against a combined protein database that included *A. simplex*, *A. pegreffii* and *A. simplex x A. pegreffii* (hybrid) protein sequences from Anisakis DB (www.anisakis.mncn.csic.es) (containing 252.756 protein coding genes included their corresponding reversed entries), using the Mascot Server v. 2.6.1 (Matrix Science, London, UK). The search parameters were enzyme, trypsin; allowed missed cleavages, 2; fixed modifications, iTRAQ4plex (N-term and K) and beta-methylthiolation of cysteine; variable modifications, oxidation of methionine, acetyl (Protein N-term), pyrrolidone from E, and pyrrolidone from Q. Peptide mass tolerance was set at ±25 ppm for precursors and 0.05 Da for fragment masses. The interval of confidence for protein identification was set to ≥95% (*p* < 0.05) although a threshold for correct identification of individual peptides was established above the 1% false discovery rate (FDR). Only proteins with at least two quantified peptides were considered in the quantitation statistical model. A 5% quantitation FDR threshold was used to consider differentially expressed proteins.

A comparison of shared and independent proteins in the iTRAQ-2 and iTRQ-3 experiment was performed on both total identified proteins and those which accomplished the criteria of 5% quantitation FDR threshold by mean of Venn diagrams using Venny software (CSIC, Madrid, Spain) [40].

#### 2.5.1. Coding Protein Regulation Values

Selection of the proteins which can be considered as taxonomic markers was done by coding the resulting quantification of proteins expression in the iTRAQ experiments to obtain significant discrete states (0, 1). Codification (0, 1) was based on level of signification of ratio (Log_2_ ratios) of the iTRAQ comparison experiments, and according to the criterion of signification for continuous characters [41,42], such that 0 is down-regulated and 1 is an up-regulated protein [35]. Only proteins that were identified in all biological replicates showing some significant values were accepted. When expression values of any protein did not follow the same tendency in both experiments, the codification considers both possibilities (up or down regulated), and it is codified as question mark (?). Selected proteins were submitted to a functional protein association networks analysis by STRING software [43].

#### 2.5.2. Statistical Comparison and Validation Experiments

In order to compare and assess the biologically independent pools of the two quantitative experiments iTRAQ2 (*A. simplex*-1, *A. pegreffii*-1, *A. simplex*-2, and Hybrid-1) and iTRAQ3 (*A. simplex*-3, *A. pegreffii*-2, *A. simplex*-4 and Hybrid-2), correspondence analysis was performed for proteins whose Log_2_ ratio values were obtained for both experiments according the established value thresholds. The final result of codified proteins was assessed by means of principal component analysis (PCA) to establish the overall resemblance of *A. simplex*, *A. pegreffii*, and their hybrids. For both multivariate exploratory methods, Statistica v.6 programme (Tulsa, OK, USA) was used [44].

## 3. Results

**Figure 1:**
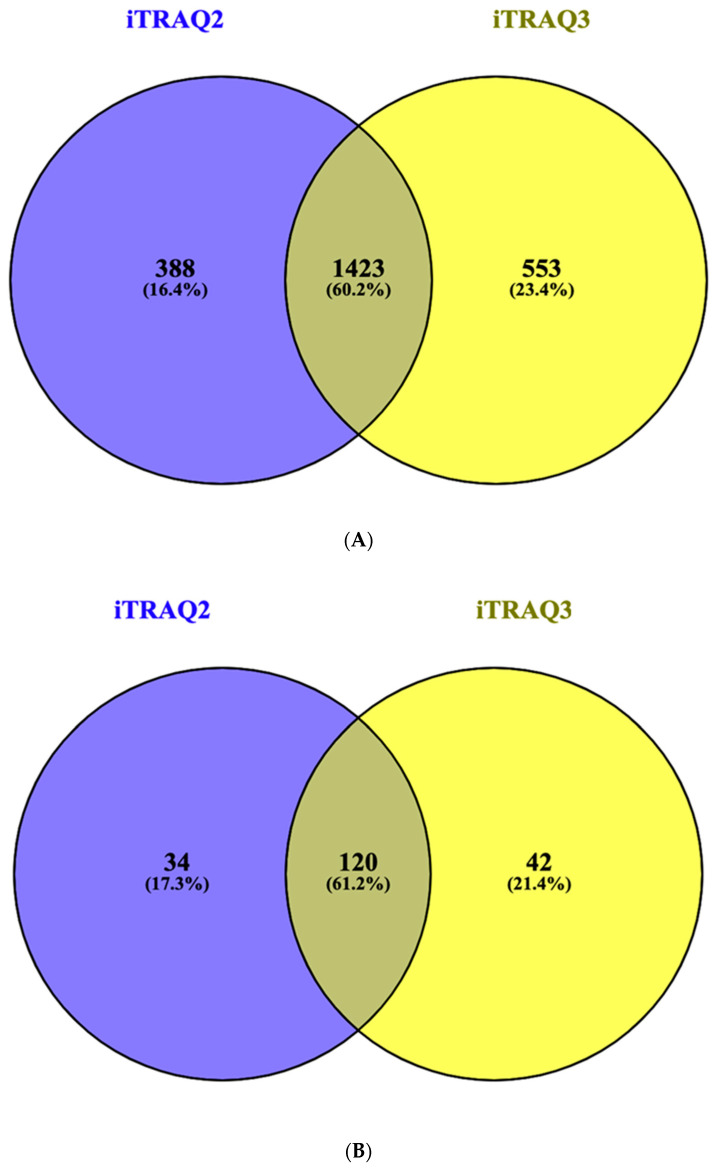
Overall comparison between both iTRAQ experiments as criteria of validation. (**A**) Venn diagram of total identified proteins comparing both experiments. (**B**) Venn diagram of proteins according to established significant criterion of false discovery rate (FDR) value (*p* < 0.005).

The biological samples are compared by means of two different iTRAQ experiments (iTRAQ2 and iTRAQ3) following the labelling scheme of Supplementary File 1. All comparisons were performed always using an independent *A. simplex* as a reference. Consequently, for iTRAQ2 the comparison was: *A. pegreffii*-1 vs. *A. simplex*-1, Hybrid-1 vs. *A.simplex*-2, and *A. simplex*-2 vs. *A. simplex*-1. For iTRAQ3, the comparison was: *A. pegreffii*-2 vs. *A. simplex*-3, Hybrid-2 vs. *A. simplex*-4, and *A. simplex*-4 vs. *A. simplex*-3. In total 1811 and 1976 proteins were respectively identified by both iTRAQ experiments (Supplementary File 1) however both experiments share 1423 common proteins (Figure 1A).

Criterion for identification of differential regulation among them, were at least two peptides showing a 95% level of signification (*p* < 0.05) of differentially regulated proteins for 1% FDR at peptide quantitation level, measured by Log_2_ ratios of the relative protein/peptide abundances of all independent biological pools compared with their respective references (*A. simplex*-1 and *A.simplex*-2; *A. simplex*-3 and *A. simplex*-4). One hundred ninety-six proteins accomplished this criterion (Supplementary File 1, summary_quant_iTRAQ2-3); 154 proteins in iTRAQ2 and 162 proteins in iTRAQ3; both experiments share 120 proteins with significant criteria (Figure 1B). These proteins were assigned according to their accession number in the published transcriptome [25]; their description, metabolic and biological function and homology with other nematodes, when possible, are included in the Supplementary Materials (Supplementary File 1, Functional Analysis).

**Figure 2:**
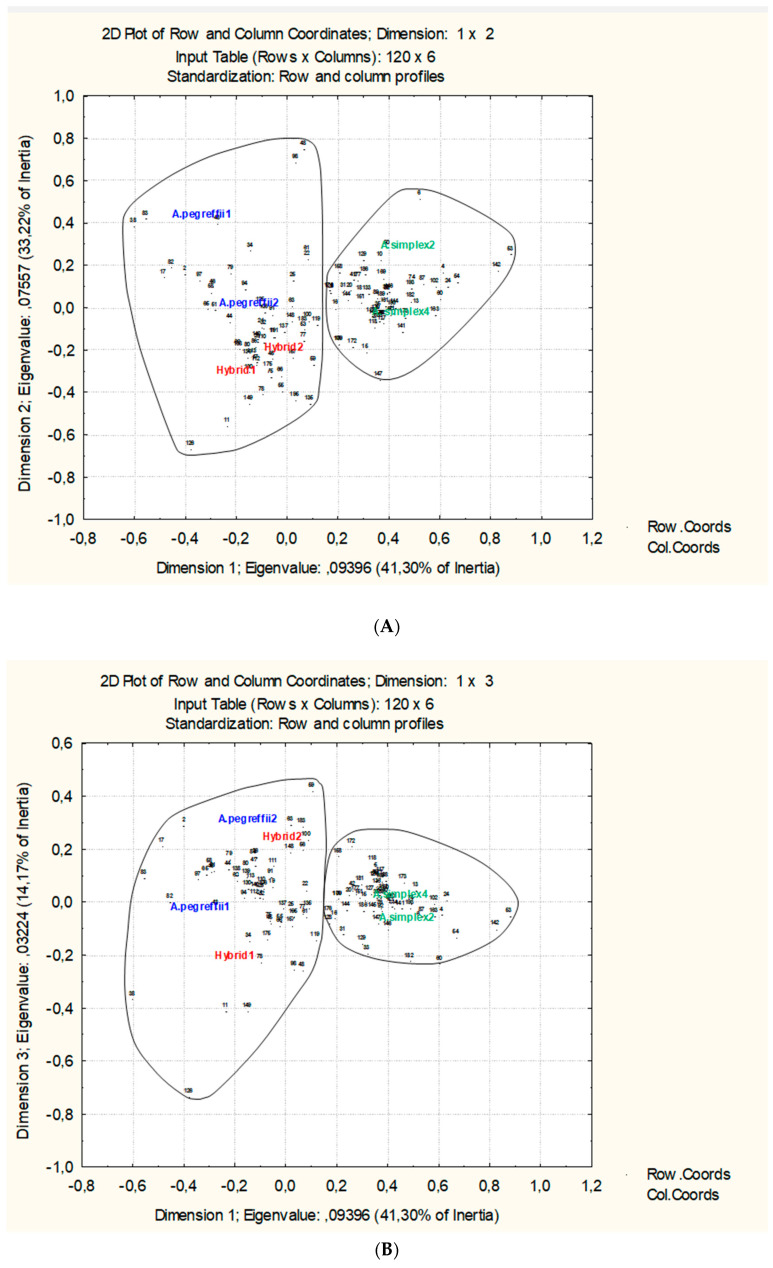
Factorial analysis of correspondence considering the 120 proteins that are common and the raw Log_2_ ratio data of statistically significant identifications according the FDR value (*p* < 0.05). Proteins and biological replicates are disposed and linked in the factorial space (**A**), Dimension 1 vs. Dimension 2; (**B**), Dimension 1 vs. Dimension 3, splitting the overall set of proteins, as possible markers of *A. simplex* and *A. pegreffiii* hybrid. Disposed variables (nematodes biological replicates) and cases (proteins) explain the 88.69% of total variability.

Analysis of correspondence considering the raw data of 196 proteins only is possible for the 120 common proteins (Figure 2) because for these there are values in both experiments and replicates. This analysis confirms the biological independence of the selected pools of L3 individuals detecting the variation in protein expression. The total variability is explained at 88.689% by the first three axes (41.30%, 33.22%, and 14.17% respectively). There are two sets of proteins that split the factorial space in those whose expression is related to *A. simplex* and other whose expression is more related to *A. pegreffii*-Hybrid (Figure 2A,B). The protein expression should be more variable in *A. pegreffii* than in *A. simplex* and Hybrid would resemble more to *A. pegreffii* due they share similar expression level in more common proteins than with *A. simplex.*

However, from these 120 common proteins, there were 24 whose level of expression is so variable (up-regulated or down-regulated) in the reference biological replicates that they cannot be considered as good taxonomic markers. In fact, given that *A. pegreffii* and the hybrid genotypes were two times separately compared with two replicates each of *A. simplex* (*A. simplex*-1 and *A. simplex*-2; *A. simplex*-3 and *A. simplex*-4) the reference ratio of Log_2_ for both *A. simplex* replicates (As2/As1 and As4/As3) approximates 1 (i.e., ratio of comparison of *A. simplex* with itself), defining the tendency of down or up regulation of proteins. In conclusion, from the first 196 proteins that showed significant values in the FDR that were not considered for conversion to binary states, those which did not show values in some of the replicates or in which the compared Log_2_ ratios (*A. simplex*-2/*A. simplex*-1 or *A. simplex*-4/*A. simplex*-3) were significant, indicating that some of the replicates deviated from the value of 1. This is because both *A. simplex* pools are from the same speciesm and the considered set of proteins have to be more similar in their expression. Consequently, 96 proteins were chosen for binary conversion of their expression level.

**Figure 3:**
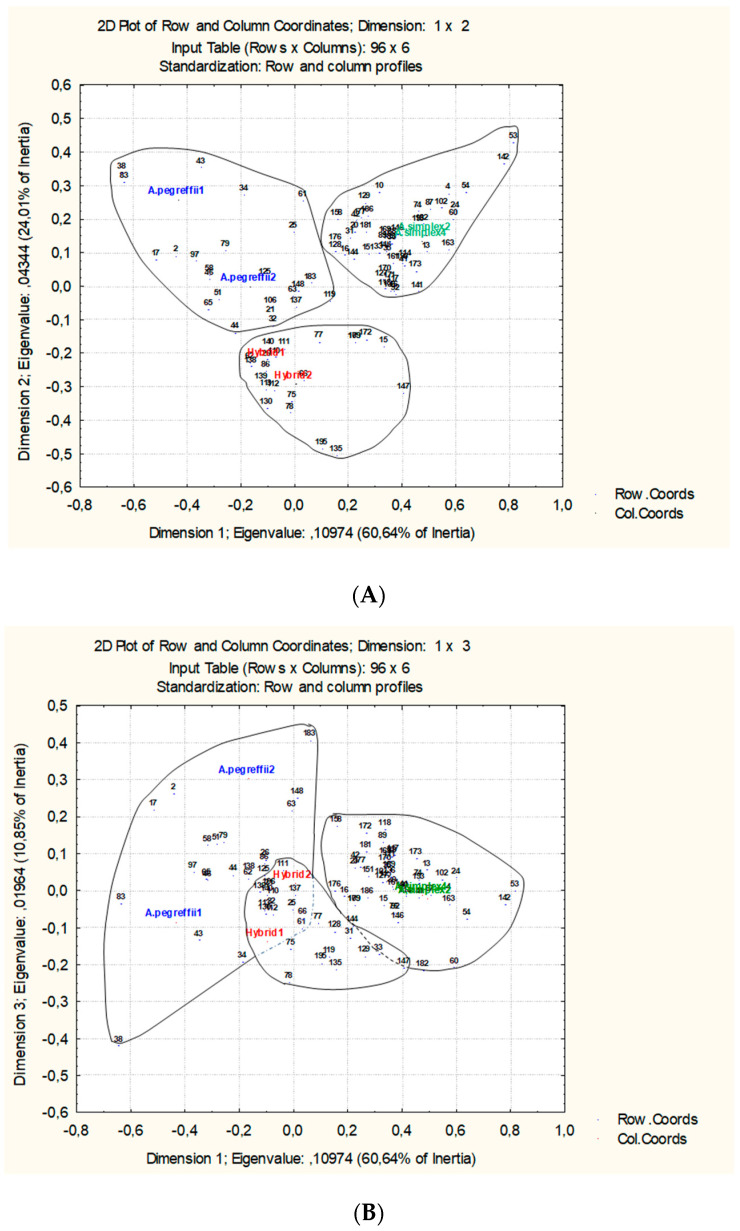
Factorial analysis of correspondence considering only the 96 proteins which do not have opposite values of expression (up-regulated or down-regulated) in the references pools of *A. simplex*. Data are the raw Log2 ratio of statistically significant identifications according the FDR value (*p* < 0.05). Proteins and biological replicates are disposed and linked in the factorial space (**A**) Dimension 1 vs. Dimension 2; (**B**) Dimension 1 vs. Dimension 3, splitting the overall set of proteins in three sets as possible markers of *A. simplex*, *A. pegreffiii*, and hybrid. Disposed variables (nematodes biological replicates) and cases (proteins) explain the 95.5% of total variability.

The correspondence analysis for these 96 proteins (Figure 3) increases the explained source of variation up to 95.5% for the first three axes (64.64%, 24.01%, and 10.85% respectively) when compared with the former correspondence analysis (Figure 2). This means that most of the detected variations could be explained by the selected proteins that form three different groups, each of them with direct relationships with the considered taxonomic units (*A. simplex*, *A. pegreffii*, and hybrid). Both species and their common hybrids are also clearly distinguished and split, narrowing the distance in the factorial space for *A. simplex* biological replicates and also for hybrids biological replicates (Figure 3A,B). The influence of the sets of proteins is reflected according their level of expression. In this step, we choose those that discriminate according significant statistical criterion.

The overall level of significance expression for these proteins was obtained considering the three Log_2_ ratios (*A. pegreffii/A. simplex*, A. hybrid/*A. simplex*, and *A. simplex*/*A. simplex*) and their relations with the values of average ($\bar{x}$) and standard deviation (δx) ratios including the biological replicates and the two experiments. According to this, the level of significance would be established by 2δx if δx is ≤ $\bar{x}$ or δx if δx ≥ $\bar{x}$ (gap-coding criterion of Archie [41]) (Table 1). This allows the conversion of the significantly regulated proteins as continuous characters in discrete or binary states (1, up-regulated protein; 0, down-regulated protein) [42,45,46].

**Figure 4:**
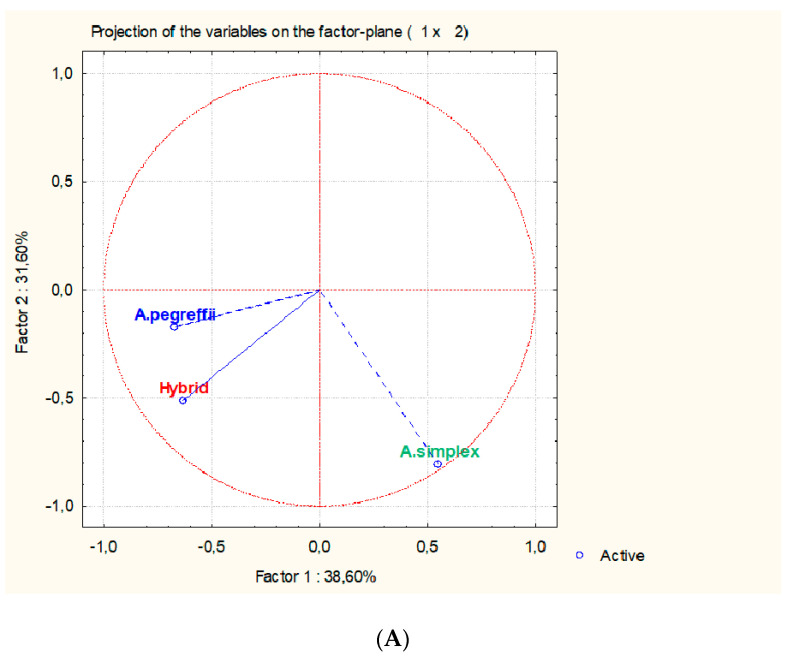

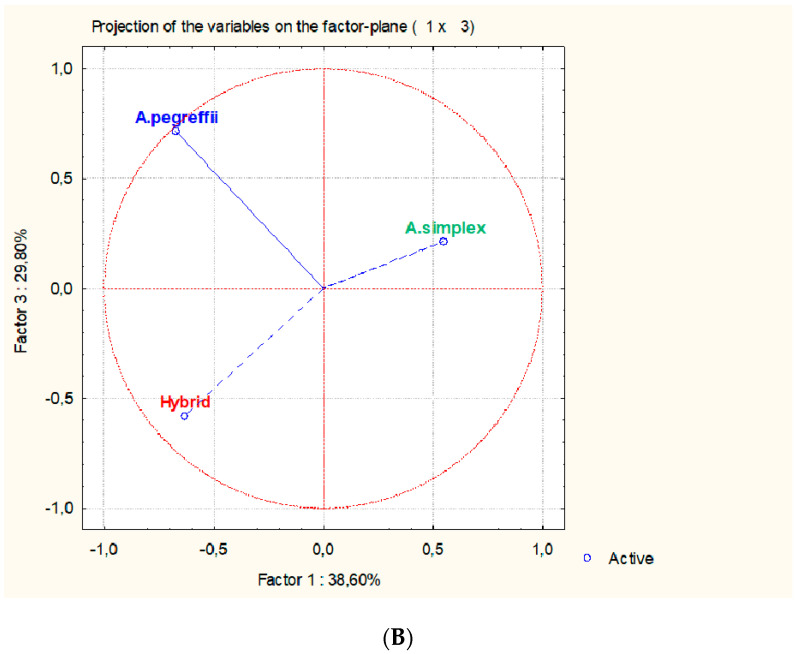
Principal component analysis of selected proteins after conversion to binary state data. Analysis confirms viability of the proposal method for conversion from quantitative to qualitative values. Contribution of proteins and correlation are in Supplementary Table S1. Percentage of explanation is for total variability is 100% for three factors (axes). (**A**) Factor 11 vs. Factor 2; (**B**) Factor 1 vs. Factor 3.

Principal component analysis (Figure 4) for the converted binary data (Table 1) confirms that hybrid genotype resembles *A. pegreffii* more than *A. simplex*. The percentage of explanation is 100%—38.60% for first factor, 31.60% for second factor, and 29.80% for third factor. This demonstrates that the chosen proteins are good taxonomical markers to differentiate among the studied species and their hybrid. The factorial space is mainly formed by the proteins which show significant discriminant values according the Archie statistical criteria [42,43] of Table 1 where at least two values (0 and 1) are undoubtedly stablished (shadow highlighted). All of them have an important contribution in factorial space defining the taxonomic differences among *A. simplex*, *A. pegreffii*, and Hybrid. Proteins 13, 61, 76, 78, 92, 119, 172, 173 and 183 have an important contribution to form the factors, but they have opposite qualitative values (up or down regulated) in one of the two experiments, being considered as ambiguous markers. Protein 42, although it notably contributes to factor 2, does not show discriminant power (Supplementary Materials, Table S1).

**Figure 5:**
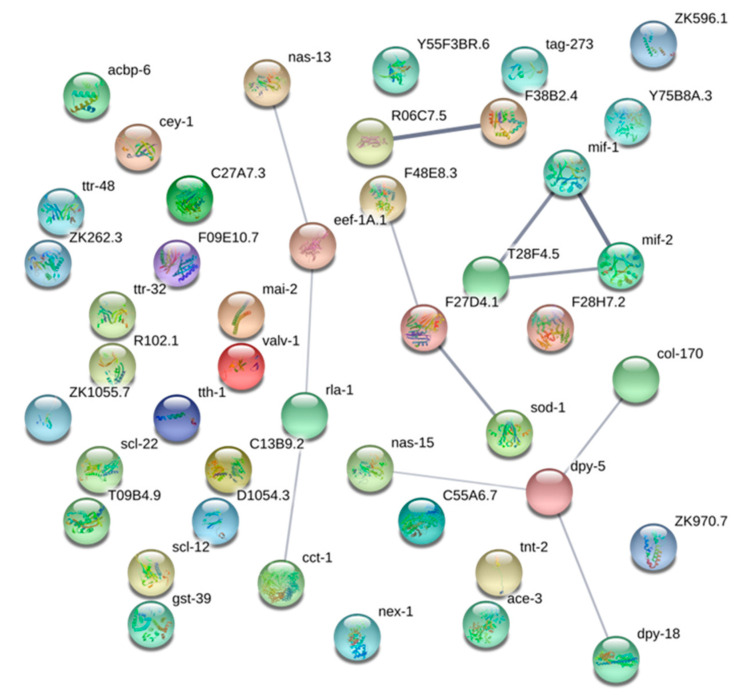
Network connection for 44 from the 96 selected proteins that accomplish the statistical criteria. *Caenorhabditis elegans* orthologs and the STRING [43] proteins names are included in Table 1.

The interaction network of the 96 selected proteins shows a very weak connection among them (Figure 5) when the confidence limit is setting to 0.7. The interaction analysis was performed using the *Caenorhabditis elegans* database of proteins of STRING. The *C. elegans* orthologs and their proteins name are referred in Table 1 and Table 2. Fourty-four nodes with 12 edges (expected 3) were detected; however, most of the nodes are considered “orphans.”

Binary conversion provided evidence that 59 proteins are not informative because they present state 1 or 0 in the three assays and ambiguity at least for the same entity (it can be 0 or 1) referred as (?) in Table 1. In total, 37 proteins are clearly significant and are candidates to be considered as protein taxonomical markers (Table 2). Only 12 of these taxonomical markers are included in the functional analysis and from them, structural protein dpy-5 (cuticle collagen) is the node of a cluster including another two proteins markers (col-170 and nas-15) and dpy-18 (procollagen-proline 4-dioxygenase activity). None of the interacting cluster formed by mif-1 (Tranitheryn5), T28F4.5 (Bm-DAP-1 identical), and mif-2 (cold-shock DNA-binding domain) can be considered taxonomical markers based on the statistical criteria. The last marker (nas-13, predicted zinc metalloase) is weakly included in a linear cluster with eef-1A.1, rla-1, and cct-1.

## 4. Discussion

Global proteomics analysis in anisakids nematodes is a very promising discipline [32,47] from clinical to diagnostic tools. There are approximately 20,000 genes in nematodes, however it is estimated that there are hundreds of thousands of proteins isoforms, most of them modified at postranscriptional stage. This complexity (proteins and isoforms) requires a very confident methodology which in our case is provided by iTRAQ approach allowing in the same experiment separation, quantification and identification. Comparing *A. simplex*, *A. pegreffii*, and their hybrid by means of two independent experiments is the basis the definition as biomarker discovery phase in a global quantitative proteomic analysis [26]. Beause quantification is very exact, the data are suceptible to statistical analysis. We have assesed the independence of the compared biological pools through multifactorial statistical methods wich order the total factorial space variability and also define the importance of proteins. The biological independence of both experiments comparing four biological replicates of *A. simplex* and two other biological replicates for *A. pegreffii* and their common hybrids has been assesed by means of multifactorial correspondance analysis (Figure 2 and Figure 3). Lastly, selected significant proteins (Table 1) that acomplish the criteria for identification (*p* < 0.05 FDR) were also submited to a direct test in order to convert the quantitative data of both experiments in qualitative one reflecting the expression protein tendence (up or down regulated) as binary data, as was formerly proposed [35].

In spite that the hybrid genotype has 50% of each parental species (Hybrid-1 from *A. simplex* cluster; Hybrid-2 from *A. pegreffii* cluster), the selected proteins markers share common expression pattern (Figure 3). With these results, it is also possible to compare by means of similarity clustering or by sequential taxonomic analysis to assign the parental species. Principal component analysis (Figure 4) confirms the binary conversion and stress the contribution of each protein marker by means of their contribution to formation of factorial space (Supplementary Materials, Table S1). Accordingly, the hybrid genotype shares 19 proteins states with *A. pegreffii*, while only 4 protein states are shared with *A. simplex*. *A. pegreffii* and *A. simplex* share five proteins states. This confirms that although the hybrid is separated from the other two, it shares more proteomic similarities with *A. pegreffii* than with *A. simplex* such as was demonstrated when allergenic proteins have been compared in [22].

The main characteristics (including the biological and biochemical process in which they are involved) and difference among the 37 proposed proteins markers among the studies taxonomic entities are summarized in Table 2. These proteins are encoded in 33 different loci. Lipase class 3 family protein may function as a lipase (GO:0006629; catalysis of the hydrolysis of ester bonds of insoluble substrates such a triglycerides), they are widely distributed in animals, plants and prokaryotes. It is upregulated in *A. pegreffii* compared with *A. simplex* and their hybrid genotype. The class 3 family proteins are distantly related to other lipase families [48].

The leucine-rich repeats-containing domain shows equal significant differences in five loci. This domain is evolutionarily conserved in many proteins associated with innate immunity in plants, invertebrates and vertebrates. Serving as a first line of defense [49] is up-regulated for the five transcripts of *A. simplex*, while it is down-regulated in *A. pegreffii* and hybrid genotype, although one of the transcripts appears as unambiguous for *A. pegreffii* in Table 1 (ANAP13823-2TR). Transthyretin 46 is a protein known as thyroid hormone-binding protein (as a probable thyroxine transporter) considered of extracellular region or secreted protein [50], but it has also been sequenced in nematodes (*Toxocara canis* and *Caenorhabditis elegans*); it is up-regulated in *A. simplex* and hybrid genotype. Zinc metalloase nas-13, Zinc metalloase nas-15, and briggsae CBR-NAS-13 are endopeptidases (GO:0008270, GO:0004222; proteolytic peptidases) [51]. The endopeptidases are a very large family of proteolytic proteins present along the whole biological evolutionary tree. This would explain how the four transcripts (in four different loci) have different representation (protein isoforms) in the three studied entities.

The allergenic proteins Ani s12 [52] and Ani s14 [53] have been demonstrated to be major allergens [54], although no functional or biological processes are known. Ani s12 is up-regulated in *A. pegreffii* and the hybrid genotype, while is down-regulated in *A. simplex*. However, Ani s14 is down-regulated in both species while is up-regulated in their hybrid. SXP RAL-2 family 2 isoform 1 is demonstrated to be the heat-stable allergen Ani s8. Within this family of proteins there is another allergen of *A. simplex* (Ani s5) [55]. This Ani s8 shares a high homology with Ani s5 having been shown the IgE cross-reactivity between both allergens [56]. This allergen codified in three different transcripts from two loci is up-regulated in *A. simplex* and down-regulated in *A. pegreffii* and the hybrid genotype. UA3-recognized partial is the *Anisakis simplex* UA3-recognized allergen Ani s7 [57]. This allergen appears as significant expression state in four transcripts from two different loci (one and three transcripts respectively). When both loci are considered, the protein is down-regulated in *A. simplex* and up-regulated in the hybrid genotypes, while for *A. pegreffii* it is up-regulated for one locus and ambiguous for the other. Allergen Ani s10 [58] is down-regulated in *A. pegreffii* and upregulated in *A. simplex*, while both expression states in the hybrids. *Anisakis simplex* Anis11L1 mRNA for Ani s11-like protein precursor, complete cds, precursor de Ani s11 [59] is only up-regulated in *A. simplex*.

Ancylostoma secreted protein is differentially expressed through five transcripts from five different loci, known as venom allergen protein; it is upregulated in *A. pegreffii* and the hybrid genotype. This type of protein is very important for plant and animal nematodes. They are secreted during several stages of parasitism as a mix of proteins with presence of a structurally conserved group of venom allergen-like proteins (VALs) [60] causing damage to host tissue, and they are considered important proteins in host parasite relationships. This protein is considered homologous of Venom allergen 5.02 codified by two different loci.

Mitochondrial import inner membrane translocase subunit TIM44 (GO:0030150) is up-regulated in the hybrid genotype and down-regulated in both species. It interacts with mtHsp70 chaperone protein (GO:0051087) and is member of the complex found in the inner mitochondrial membrane [61]. Carboxylic ester hydrolase is a member of the cholinesterases family whose function is to act as neurotransmitter [62] in the membrane. It is up-regulated in *A. pegreffii* and down-regulated in *A. simplex*, and in the hybrid genotype, it can be present in both states. The DUF4440 domain-containing protein is an uncharacterized, hypothetical protein with high homology with LOC101164525 (*Oryzias latipes*) and a hypothetical protein ASU_00001 with protein R102.1 from *C. elegans*; both proteins were sequenced for transcriptomics comparison of the tree entities [25]. Finally, there are different expression in the structural protein cuticle collagen dpy-5 (GO:0042302) (down-regulated in *A. pegreffii*, up-regulated in hybrid genotype and ambiguous in *A. simplex*); this protein encodes a cuticle procollagen and is responsible for final body phenotype [63]. There are four uncharacterized proteins (numbers 75, 97, 135, and 141) which are however statistically very representatives.

The proteomics approach presented here characterizes the level of protein expression in order to obtain robust protein markers which can be used both as a taxonomical and clinical tool due the capacity of proteomics technology to process in a rapid way a high number of samples. The proposed method is very suitable for data such as continuous values with reliable confidence such as those obtained by iTRAQ analysis [35]. These data are clear when statistically significant differences of exact and precise proteomics can be recorded and applied in a binary matrix [29,34], as is the case of this study.

In conclusion, a total 1811 and 1976 proteins were identified in the two iTRAQ experiments comparing the proteome of *A. simplex*, *A. pegreffii*, and their hybrid genotype. From those, 37 proteins are robust candidates as markers according to the confidence limits imposed by iTRAQ methodology. The statistical conversion of continuous characters (Log2 ratios of the relative protein/peptide abundances) to binary states allows us to recognize and simplify the statistical significance of proteins regulation level (upregulated or down-regulated). The proposal of these proteins as candidate biomarkers is based on the quantitative identification of proteins combined with a statistical approach (multidimensional and classical parametric) used to reinforce the selection, however a good marker requires further development in several predictive tests. The taxonomy of the genus *Anisakis* is solidly founded avoiding environmental and fish host effects for species diagnosis. Consequently, a confirmatory analysis to demonstrate that protein selection accomplished the defined criteria regarding expression is coincident with the molecular taxonomy, which will allow us to develop high-throughput methodologies applied to clinical, epidemiological, and food technology. The development of proteomic technologies has greatly accelerated the confirmation of potential proteins as biomarkers. Quantitation using multiple-reaction monitoring mass spectrometry (MRM-MS) in combination with isotope-labeled internal standards has driven what is known as targeted proteomics [64], focused on targeted acquisition and targeted data analysis applying mass spectrometry [65]. Accordingly, the experimental demonstration to validate the taxonomical biomarkers we have proposed in this study has to be developed closing the canonical approach for biomarker detection using proteomics [66].

## Figures and Tables

**Table 1:** Ninety-six selected candidates among the differential proteins for 5% false discovery rate (FDR) at quantitative level of signification and codification in binary states according the statistical criterion of Archie [42].

	*A. pegreffii*/*A. simplex*		A. hybrid/*A. simplex*		*A. simplex*/*A. simplex*		Binary States (0), Downregulated; (1), Upregulated; No Differences in Regulation)					*C. elegans* Ortholog	STRING Proteins
	Replicate_1 (iTRAQ-2) *A. pegreffii*-1 vs. *A. simplex*-1	Replicate_2 (iTRAQ-3) *A. pegreffii*-2 vs. *A. simplex*-3	Replicate_1 (iTRAQ-2) Hybrid1 vs. *A. simplex*-2	Replicate_2 (iTRAQ-3) Hybrid2 vs. *A. simplex*-4	Replicate_1 (iTRAQ-2) *A. simplex*-2 vs. *A. simplex*-1	Replicate_2 (iTRAQ-3) *A. simplex*-4 vs. *A. simplex*-3	Ratio Average	Standard Deviation	*A. pegreffii*	Hybrid genotype	*A. simplex*		
Protein-AC	Ratio	Ratio	Ratio	Ratio	Ratio	Ratio							
2. ANAH10496_4TR	**5.11**	**4.44**	**2.38**	**2.46**	0.78	0.91	2.680675	1.784102	**1**	**0**	**0**	Q9XTR8	ZK262.3
4. ANAH1227_2TR	**0.49**	**0.42**	**0.40**	**0.42**	1.18	1.03	0.658703	0.350961	**0**	**0**	**1**		
10. ANAH1517_2TR	0.98	0.69	**0.49**	**0.60**	1.25	1.05	0.842266	0.293623	1	?	1		
13. ANAH1652_3TR	**0.38**	**0.53**	**0.52**	**0.60**	0.96	1.00	0.663876	0.255699	?	1	1	O17389	tth-1
15. ANAH19571_1TR	**0.40**	**0.58**	1.11	1.10	1.00	0.94	0.855733	0.296167	**0**	**1**	**1**	Q23378	ttr-48
16. ANAH1985_1TR	0.93	0.82	1.12	**0.59**	0.93	1.11	0.913812	0.198826	1	1	1	Q10576	dpy-18
17. ANAH2130_3TR	**7.92**	**6.63**	**4.50**	**3.04**	0.88	0.92	3.981989	2.920965	**1**	**0**	**0**	Q20191	nas-13
20. ANAH23051_1TR	0.97	0.91	0.85	**0.62**	1.06	1.14	0.925959	0.180845	1	1	1		
21. ANAH23235_2TR	1.63	1.41	**1.98**	1.32	0.89	0.96	1.366078	0.412306	1	1	1		
24. ANAH24424_1TR	**0.38**	**0.44**	**0.37**	**0.47**	1.06	1.05	0.628614	0.331899	**0**	**0**	**1**		
25. ANAH276_4TR	**1.90**	1.04	1.31	0.92	1.08	1.21	1.243492	0.348480	1	1	1	H2L2L1	tag-273
26. ANAH2918_2TR	**1.91**	**2.03**	**2.37**	**2.60**	1.08	1.00	1.832552	0.658529	**1**	**1**	**0**		
30. ANAH3118_1TR	0.69	**0.59**	0.62	0.73	1.12	1.00	0.791315	0.217149	1	1	1	Q23545	ZK596.1
31. ANAH3489_5TR	1.07	**0.53**	1.00	**0.65**	1.05	1.06	0.892934	0.238308	1	1	1	P41988	cct-1
32. ANAH3571_1TR	1.76	1.26	**2.05**	1.63	0.92	1.01	1.439694	0.444919	1	1	1		
33. ANAH357_3TR	0.80	**0.39**	1.01	**0.62**	1.28	0.83	0.819474	0.305428	?	1	1		
34. ANAH3624_1TR	**3.11**	0.93	1.66	0.86	1.12	1.18	1.477237	0.847699	?	0	0	P34697	sod-1
35. ANAH4078_4TR	0.61	0.74	0.79	**0.63**	0.99	1.13	0.815939	0.205690	1	1	1	Q22850	T28F4.5
38. ANAH4433_8TR	**11.21**	1.68	**5.98**	1.01	1.00	1.11	3.666057	4.166937	?	0	0		
41. ANAH5032_5TR	**0.45**	**0.51**	0.68	**0.59**	0.90	0.94	0.679135	0.205099	1	1	1	Q17522	val-1
42. ANAH5172_1TR	0.91	0.83	0.66	**0.57**	1.03	0.98	0.831024	0.180319	1	1	1	Q21774	R06C7.5
43. ANAH5273_1TR	**4.23**	1.22	1.31	1.20	0.94	1.14	1.673703	1.260709	?	0	0		
44. ANAH6141_1TR	**2.88**	**2.27**	**2.72**	**2.66**	1.08	0.91	2.087151	0.871078	**1**	**1**	**0**		
46. ANAH6329_2TR	**3.93**	**2.36**	**2.40**	**2.37**	1.04	0.93	2.171381	1.098905	**1**	**1**	**0**	P55115	nas-15
51. ANAH7542_3TR	**3.06**	**2.86**	**2.77**	**1.82**	0.91	0.95	2.060886	0.976528	**1**	**?**	**0**		
53. ANAH777_1TR	**0.30**	**0.25**	**0.13**	**0.25**	1.04	1.01	0.497237	0.412228	**0**	**0**	**1**		
54. ANAH777_4TR	**0.38**	**0.29**	**0.43**	**0.30**	1.00	1.01	0.568653	0.344521	**0**	**0**	**1**	A0A078BPG0	F09E10.7
58. ANAH9144_1TR	**3.84**	**3.10**	**2.70**	**2.00**	1.03	1.03	2.285082	1.139551	**1**	**?**	**0**		
60. ANAH9523_1TR	**0.39**	**0.15**	**0.58**	**0.33**	0.89	0.98	0.554457	0.327235	**0**	**0**	**1**	P90781	C55A6.7
61. ANAH9572_5TR	**1.98**	0.78	0.98	0.88	1.30	1.08	1.167379	0.439292	?	1	1		
62. ANAH9579_2TR	**2.09**	**2.07**	**2.94**	**2.32**	0.90	0.97	1.880837	0.798899	**1**	**1**	**0**	Q09567	F48E8.3
63. ANAH964_1TR	1.52	1.72	1.00	**1.76**	1.06	1.05	1.351465	0.357845	1	1	1	A0A0K3AST9	scl-22
65. ANAP10737_1TR	**3.95**	**2.73**	**3.17**	**2.70**	0.91	1.01	2.413920	1.212109	**1**	**1**	**0**	O02161	T09B4.9
66. ANAP1108_1TR	0.96	1.23	**2.65**	1.42	0.88	1.12	1.377658	0.652672	**0**	**1**	**0**		
74. ANAP13823_2TR	**0.55**	**0.53**	**0.48**	**0.46**	1.04	1.01	0.676710	0.269445	**?**	**0**	**1**		
75. ANAP14229_1TR	1.34	1.03	**3.00**	**2.25**	0.92	1.16	1.617101	0.827149	**0**	**1**	**0**		
76. ANAP16339_1TR	**0.53**	**0.46**	0.80	0.85	1.04	0.92	0.766507	0.225415	?	1	1		
77. ANAP16888_4TR	1.13	0.91	**1.87**	1.50	1.16	1.00	1.260748	0.360601	1	1	1		
78. ANAP17930_1TR	1.20	0.93	**3.67**	**1.78**	0.81	1.20	1.598792	1.069051	0	?	0	Q9U295	ace-3
79. ANAP1821_4TR	**3.18**	**2.37**	1.70	1.67	1.20	0.85	1.827840	0.837408	**1**	**?**	**0**		
83. ANAP228_1TR	**10.84**	**4.02**	**3.05**	**3.07**	0.82	0.98	3.795073	3.676560	?	0	0		
86. ANAP245_9TR	1.84	**1.96**	**2.42**	**2.69**	1.07	0.92	1.816920	0.710125	**1**	**1**	**0**		
87. ANAP258_1TR	**0.51**	**0.45**	**0.49**	**0.43**	1.05	1.03	0.660602	0.294249	**0**	**0**	**1**		
88. ANAP258_2TR	0.62	0.70	**0.55**	**0.64**	1.00	1.03	0.757335	0.207204	1	1	1		
89. ANAP258_4TR	0.62	0.84	0.63	**0.61**	1.00	1.03	0.788300	0.196369	1	1	1		
92. ANAP293_7TR	**0.52**	**0.48**	0.86	0.86	1.14	0.86	0.787651	0.249029	?	1	1		
97. ANAP3585_1TR	**4.09**	**2.62**	**2.55**	**1.83**	0.93	0.87	2.149391	1.215058	**1**	**?**	**0**		
102. ANAP4471_3TR	**0.43**	**0.45**	**0.40**	**0.42**	0.98	1.04	0.620592	0.301599	**0**	**0**	**1**	E3W744	C27A7.3
106. ANAP5435_12TR	**1.93**	1.48	**1.90**	1.64	1.19	0.86	1.499992	0.415442	1	1	1		
109. ANAP6590_1TR	**0.59**	0.65	1.17	1.14	0.97	0.84	0.893992	0.243219	1	1	1		
110. ANAP667_10TR	1.70	1.44	**2.30**	**2.14**	1.03	0.96	1.595647	0.559270	**1**	**1**	**0**		
111. ANAP667_11TR	1.56	1.56	**1.93**	**2.11**	1.11	0.96	1.537201	0.448659	1	1	1		
112. ANAP667_16TR	1.48	1.34	**2.69**	**2.22**	0.90	0.90	1.589128	0.727740	**?**	**1**	**0**		
113. ANAP667_20TR	1.74	1.38	**2.57**	**2.66**	0.94	0.86	1.692057	0.785112	**?**	**1**	**0**	Q20140	F38B2.4
114. ANAP67_16TR	**0.53**	**0.54**	0.72	0.67	1.08	1.02	0.760630	0.236209	1	1	1		
117. ANAP770_2TR	**0.45**	0.71	0.69	0.79	0.90	1.04	0.763521	0.200851	1	1	1	Q18785	mif-2
118. ANAP7903_1TR	**0.44**	0.91	0.75	0.80	0.91	1.07	0.812855	0.215385	1	1	1	G5EEA8	nex-1
119. ANAP791_1TR	1.09	**0.56**	1.51	0.98	1.00	1.03	1.027052	0.303891	?	1	1	Q9NAB0	gst-39
125. ANAP975_1TR	**2.00**	1.61	**1.82**	1.22	0.96	1.10	1.452455	0.420447	1	1	1		
127. ANAS1021_17TR	**0.55**	0.61	0.75	0.75	0.94	0.92	0.751676	0.156521	1	1	1		
128. ANAS1030_2TR	1.09	**0.56**	0.97	0.76	0.99	0.92	0.880618	0.193530	1	1	1		
129. ANAS1197_1TR	1.13	**0.35**	0.75	**0.59**	1.26	0.90	0.830480	0.338069	?	?	1		
130. ANAS12312_1TR	1.59	1.44	**2.94**	**2.67**	0.82	0.93	1.732642	0.886435	**0**	**1**	**0**		
135. ANAS163_8TR	0.60	**0.44**	**2.36**	**2.53**	0.95	1.04	1.319332	0.899014	**0**	**1**	**0**	O62213	cey-1
136. ANAS1679_11TR	**0.45**	**0.58**	0.69	0.77	0.90	0.87	0.709232	0.174788	1	1	1	A0A1I6CMC9	Y55F3BR.6
137. ANAS1685_8TR	1.33	1.24	**1.85**	1.05	0.85	1.17	1.248270	0.338370	1	1	1		
138. ANAS1813_1TR	**2.08**	**2.16**	**2.93**	**2.45**	0.91	0.98	1.919019	0.812140	**1**	**1**	**0**	Q9N5N3	scl-12
139. ANAS1813_2TR	**1.88**	1.76	**2.82**	**2.68**	0.89	1.02	1.840418	0.804574	**1**	**1**	**0**		
140. ANAS18294_1TR	1.78	1.55	**2.39**	**1.95**	1.01	0.95	1.604383	0.559189	**1**	**1**	**0**		
141. ANAS18621_6TR	**0.33**	**0.50**	0.79	0.69	0.95	1.02	0.714755	0.264832	**0**	**1**	**1**	Q23683	ZK970.7
142. ANAS188_11TR	**0.30**	**0.26**	**0.26**	**0.25**	1.03	1.09	0.532123	0.407959	**0**	**0**	**1**	Q86NE3	col-170
144. ANAS19190_3TR	1.01	**0.53**	0.89	0.90	0.97	1.11	0.900457	0.199744	1	1	1	Q18949	D1054.3
145. ANAS1945_1TR	0.63	0.76	0.93	**0.51**	0.87	1.31	0.836623	0.280022	1	?	1	O44441	mai-2
146. ANAS1981_2TR	0.71	**0.47**	0.78	**0.58**	1.16	1.04	0.790438	0.266683	?	1	1	P91285	dpy-5
147. ANAS2128_5TR	**0.24**	**0.19**	1.15	1.22	0.87	0.90	0.761490	0.447108	**0**	**1**	**?**	Q95Y92	ttr-32
148. ANAS2152_3TR	1.20	**1.96**	1.44	1.05	0.94	1.18	1.295756	0.368101	1	1	1	Q9U228	mif-1
150. ANAS2269_8TR	0.73	0.73	0.79	**0.66**	0.97	1.01	0.814381	0.143553	1	1	1		
158. ANAS2659_2TR	1.21	1.20	**0.59**	0.86	1.17	1.11	1.023444	0.249913	1	1	1	Q09235	C13B9.2
161. ANAS2998_4TR	0.64	**0.59**	0.69	0.83	1.06	1.09	0.816894	0.215097	1	1	1	Q21887	R102.1
163. ANAS3657_3TR	**0.34**	**0.40**	**0.61**	**0.55**	0.98	1.16	0.672240	0.330083	**0**	**0**	**1**	Q93615	F27D4.1
169. ANAS4205_3TR	0.73	0.75	**0.55**	0.76	1.33	0.83	0.824435	0.264690	1	1	1	Q7Z072	tnt-2
170. ANAS422_5TR	**0.58**	0.72	0.71	0.84	1.01	1.03	0.815319	0.178573	1	1	1		
171. ANAS422_6TR	**0.51**	0.72	0.71	0.83	0.98	1.04	0.797374	0.192933	1	1	1	Q19890	F28H7.2
172. ANAS424_11TR	**0.41**	0.89	0.81	1.13	0.81	0.92	0.829663	0.238560	?	1	1		
173. ANAS4400_1TR	**0.41**	0.71	0.68	0.77	1.10	1.13	0.801061	0.273388	?	1	1	O76449	ZK1055.7
176. ANAS5415_1TR	1.03	0.85	1.02	**0.61**	1.09	0.90	0.915958	0.177027	1	1	1		
177. ANAS543_3TR	0.94	0.84	0.70	**0.58**	1.06	1.07	0.863539	0.199145	1	1	1	Q9XW75	Y75B8A.3
179. ANAS5529_1TR	**0.59**	0.65	1.17	1.14	0.97	0.84	0.893992	0.243219	1	1	1	P91913	rla-1
181. ANAS623_3TR	0.88	0.97	0.77	**0.65**	1.05	1.24	0.925777	0.208788	1	1	1		
182. ANAS6350_1TR	**0.54**	**0.19**	0.69	**0.38**	0.84	1.07	0.616636	0.316442	**0**	**?**	**1**		
183. ANAS651_15TR	0.92	**2.33**	1.25	0.85	0.89	1.30	1.256519	0.560404	?	?	?		
186. ANAS7239_1TR	1.02	0.68	0.75	**0.64**	1.23	1.06	0.896235	0.240170	1	1	1		
189. ANAS7857_1TR	0.61	0.68	0.68	**0.58**	1.01	1.02	0.764980	0.199163	1	1	1	P53013	eef-1A.1
190. ANAS8_364TR	**0.52**	**0.54**	0.69	0.69	1.03	0.99	0.745290	0.218965	1	1	1	Q9XXJ2	acbp.6
193. ANAS9234_1TR	**0.55**	**0.58**	**0.60**	**0.53**	1.19	1.03	0.746691	0.289333	?	?	1		
195. ANAS9683_5TR	0.73	0.66	**2.75**	**2.51**	1.05	0.98	1.446199	0.930450	**0**	**1**	**0**	Q9XTR8	ZK262.3

**Table 2:** Selected differentially expressed proteins as biomarker value after comparison and conversion in binary states. For description and characteristics, the proteins sequences were subjected to blast analysis against UniProt and National Center for Biotechnology Information (NCBI) databases (1, up-regulated; 0. Down-regulated? ambiguous).

Sequence Name	*A. pegreffii*	Hybrid genotype	*A. simplex*	Seq. Length	Blast Hit Description	Blast Hit Accession UniPrtot	Top-Hit Specie	Blast E-Value	Blast Hit Score	Hit Align Length	Hit Positives	Similarity (%)	Function	Biological Process	Gene Name	*C. elegans* Ortholog	STRING Protein
2. ANAH10496_4TR	**1**	**0**	**0**	269	class 3 family-containing	KHN81003	*Toxocara canis*	2.43 × 10^−75^	237	276	178	64	hydrolase activity	Lipid metabolic process GO:0006629	ZK262.3 Tcan_15614	Q9XTR8	ZK262.3
4. ANAH1227_2TR	**0**	**0**	**1**	93	Putative leucine-rich repeat-containing protein	KHN76765	*Toxocara canis*	8.29 × 10^−38^	138	91	76	84			Tcan_08746		
15. ANAH19571_1TR	**0**	**1**	**1**	165	Transthyretin 46	KHN81755	*Toxocara canis*	6.57 × 10^−74^	213	128	112	88			ttr-46 Tcan_15247	Q23378	ttr-48
17. ANAH2130_3TR	**1**	**0**	**0**	323	Predicted Zinc metalloase nas-13	KHN70807	*Toxocara canis*	5.53 × 10^−76^	239	290	173	60	Zinc ion binding, metalloendopeptidase activity GO:0008270 GO:0004222		nas-13 Tcan_18910	Q20191	nas-13
24. ANAH24424_1TR	**0**	**0**	**1**	80	Putative leucine-rich repeat-containing protein	KHN76765	*Toxocara canis*	5.82 × 10^−19^	83	67	53	79			Tcan_08746		
26. ANAH2918_2TR	**1**	**1**	**0**	1403	Ani s12 allergen	ABL77410	*Anisakis simplex*	2.39 × 10^−78^	287	1199	467	39					
44. ANAH6141_1TR	**1**	**1**	**0**	122	Ancylostoma secreted (allergen)	KHN71039	*Toxocara canis*	4.22 × 10^−45^	154	127	90	71			ASP Tcan_02573		
46. ANAH6329_2TR	**1**	**1**	**0**	292	Ancylostoma secreted (allergen)	KHN71039	*Toxocara canis*	2.65 × 10^−60^	201	223	130	58			ASP Tcan_02573		
51. ANAH7542_3TR	**1**	**?**	**0**	336	Zinc metalloase nas-15	KHN78596	*Toxocara canis*	1.28 × 10^−73^	234	284	172	61	Zinc ion binding, metalloendopeptidase activity, GO:0008270 GO:0004222		nas-15 Tcan_05459	P55115	nas-15
53. ANAH777_1TR	**0**	**0**	**1**	101	SXP/RAL-2 family protein 2 isoform 9 (Ani s8)	BAF75711	*Anisakis simplex*	1.61 × 10^−34^	119	84	84	100			Ani s 8-9		
54. ANAH777_4TR	**0**	**0**	**1**	91	SXP RAL-2 family 2 isoform 2 (Ani s8)	BAF75704	*Anisakis simplex*	1.01 × 10^−32^	114	56	56	100			Ani s 8-2		
58. ANAH9144_1TR	**1**	**?**	**0**	151	DUF4440 domain-containing protein	A0A0M3KE35	*Ascaris suum*	1.75 × 10^−17^	79	111	64	58			ASIM_LOCUS18633	A0A078BPG0	F09E10.7
60. ANAH9523_1TR	**0**	**0**	**1**	134	Anisakis simplex Ani s11 L1 mRNA for Ani s11-like protein precursor, complete cds										(NCBI: VDK68784.1)		
62. ANAH9579_2TR	**1**	**1**	**0**	126	Venom allergen 5.02	KHN88210	*Toxocara canis*	1.01 × 10^−35^	126	118	81	69			Tcan_09440		
65. ANAP10737_1TR	**1**	**1**	**0**		Venom allergen 5.02	A0A0B2W4Q4	*Toxocara canis*	1.1 × 10^−63^	521	225	142	44			Tcan_09440	A0A0K3AST9	scl-22
66. ANAP1108_1TR	**0**	**1**	**0**	465	Mitochondrial import inner membrane translocase, subunit TIM44	A0A0M3JR34	*Ascaris suum*	0.00 × 10^0^	771	465	426	92	chaperone binding GO:0051087	protein import into mitochondrial matrix GO:0030150	ASIM_LOCUS9900	O02161	T09B4.9
74. ANAP13823_2TR	**?**	**0**	**1**	241	Putative leucine-rich repeat-containing protein	KHN76765	*Toxocara canis*	1.15 × 10^−77^	259	241	182	76			Tcan_08746		
75. ANAP14229_1TR	**0**	**1**	**0**	92													
79. ANAP1821_4TR	**1**	**?**	**0**	566	Carboxylic ester hydrolase	A0A044VHF4	*Ascaris suum Toxocara canis*	0.00 × 10^0^	713	555	422	76			(ace-4)	Q9U295	ace-3
86. ANAP245_9TR	**1**	**1**	**0**	1233	UA3-recognized partial (Ani s7)	ABL77410	*Anisakis simplex*	2.29 × 10^−72^	267	1059	430	41			(ASIM_LOCUS13453)		
87. ANAP258_1TR	**0**	**0**	**1**	119	Putative leucine-rich repeat-containing protein	KHN76765	*Toxocara canis*	5.13 × 10^−56^	191	118	102	86			Tcan_08746		
97. ANAP3585_1TR	**1**	**?**	**0**	189	Uncharacterized protein (predicted)	ERG80299A0A0M3J3R8	*Ascaris suum Anisakis simplex*	2.67 × 10^−90^	282	187	166	89			(ASIM_LOCUS2050)		
102. ANAP4471_3TR	**0**	**0**	**1**	88	Putative leucine-rich repeat-containing protein	KHN76765	*Toxocara canis*	8.20 × 10^−31^	117	88	72	82			Tcan_08746		
110. ANAP667_10TR	**1**	**1**	**0**	1279	UA3-recognized partial (Ani s7)	ABL77410	*Anisakis simplex*	5.58 × 10^−84^	302	1015	431	42			ASIM_LOCUS2158)		
112. ANAP667_16TR	**?**	**1**	**0**	1234	UA3-recognized partial (Ani s7)	ABL77410	*Anisakis simplex*	7.36 × 10^−88^	313	1199	477	40			(ASIM_LOCUS2158)		
113. ANAP667_20TR	**?**	**1**	**0**	535	UA3-recognized partial (Ani s7)	ABL77410	*Anisakis simplex*	1.11 × 10^−42^	168	596	249	42			(ASIM_LOCUS2158)		
130. ANAS12312_1TR	**0**	**1**	**0**	532	Ani s14 allergen	BAT62430	*Anisakis simplex*	1.09 × 10^−143^	416	217	217	100			(ASIM_LOCUS4926)		
135. ANAS163_8TR	**0**	**1**	**0**	88											(ASIM_LOCUS14166)		
138. ANAS1813_1TR	**1**	**1**	**0**	137	Ancylostoma secreted (allergen)	KHN88210	*Toxocara canis*	1.13 × 10^−39^	136	137	90	66			Tcan_09440		
139. ANAS1813_2TR	**1**	**1**	**0**	225	Ancylostoma secreted (allergen)	KHN88210	*Toxocara canis*	1.06 × 10^−63^	201	237	145	61			Tcan_09440	Q9N5N3	scl-12
140. ANAS18294_1TR	**1**	**1**	**0**	287	Zinc metalloase nas-15	KHN79293	*Toxocara canis*	7.98 × 10^−66^	216	231	146	63	zinc ion binding, metalloendopeptidase activity GO:0008270 GO:0004222		nas-15 Tcan_02995		
141. ANAS18621_6TR	**0**	**1**	**1**	98													
142. ANAS188_11TR	**0**	**0**	**1**	150	SXP RAL-2 family 2 isoform 1 (Ani s8)	BAF75705	*Anisakis simplex*	1.69 × 10^−70^	212	134	133	99			Ani s 8-3	Q23683	ZK970.7
147. ANAS2128_5TR	**0**	**1**	**?**	148	Cuticle collagen dpy-5	KHN71547	*Toxocara canis*	7.80 × 10^−29^	106	87	83	95	structural constituent of cuticle GO:0042302		dpy-5 Tcan_02187	P91285	dpy-5
163. ANAS3657_3TR	**0**	**0**	**1**	121	hypothetical protein ASU_00001 (DUF4440)	ERG87808	*Ascaris suum C, ekegans*	1.93 × 10^−47^	152	120	94	78			(ASIM_LOCUS1832) R102.1	Q21887	R102.1
182. ANAS6350_1TR	**0**	**?**	**1**	84	Allergen Ani s10		*A. simplex*								ASIM_LOCUS19307		
195. ANAS9683_5TR	**0**	**1**	**0**	217	briggsae CBR-NAS-13	XP_002645932	*Caenorhabditis briggsae*	4.79 × 10^−58^	192	176	126	72	zinc ion binding, metalloendopeptidase activity GO:0008270 GO:0004222		Cbr-nas-13		

## Supplementary Materials

The following are available online at https://www.mdpi.com/2073-4425/11/8/913/s1, Table S1. Proteins contribution to the factors according to the Lg_2_ ratio and correlation of studied nematodes with factors of principal component analysis (Figure 4); Supplementary File 1. Parameters of total (1811 and 1976) identified proteins and their accessions according Anisakis DB (www.anisakis.mncn.csic.es), including summary results for the 196 significant proteins (196), their functional analysis, labelling scheme, and results for the independents experiments iTRAQ-2 and iTRAQ-3.
