# Supplementary material for: Quantitative Proteomics Comparison of Total Expressed Proteomes of Anisakis simplex Sensu Stricto, A. pegreffii, and Their Hybrid Genotype
Source: Genes (Basel). 2020 Aug 10;11(8):913. doi: 10.3390/genes11080913 (PMC7465371; doi:10.3390/genes11080913)
Supplement: Supplementary file 1 [file genes-11-00913-s001.zip › Table S1.docx]

**Table S1.** Proteins contribution to the factors according to the Lg_2_ ratio and correlation of studied nematodes with factors of principal component analysis (Figure 4). Yellow rows are the proteins which show significant discriminant values according the Archy statistical criteria (Table 1); all of them have important contribution in factorial space defining the taxonomic differences among *A. simplex*, *A. pegreffii* and Hybrid. Proteins 13, 61, 76, 78, 92, 119, 172, 173, and 183 have important contribution to form some of the factors but show opposite values in some of both experiments, being considered ambiguous (?). Protein 42 although notably contributes to factor 2 does not show discriminant power.

| Protein Number | Contribution Factor 1 | Contribution Factor 2 | Contribution Factor 3 |
| --- | --- | --- | --- |
| **2** | 0.011746 | 0.951852 | 0.036401 |
| **4** | 0.940407 | 0.059275 | 0.000318 |
| **10** | 0.173401 | 0.462347 | 0.364252 |
| **13** | 0.193390 | 0.162116 | 0.644494 |
| **15** | 0.644265 | 0.029003 | 0.326732 |
| **16** | 0.524330 | 0.475490 | 0.000181 |
| **17** | 0.011746 | 0.951852 | 0.036401 |
| **20** | 0.524330 | 0.475490 | 0.000181 |
| **21** | 0.524330 | 0.475490 | 0.000181 |
| **24** | 0.940407 | 0.059275 | 0.000318 |
| **25** | 0.524330 | 0.475490 | 0.000181 |
| **26** | 0.161148 | 0.741928 | 0.096924 |
| **30** | 0.524330 | 0.475490 | 0.000181 |
| **31** | 0.524330 | 0.475490 | 0.000181 |
| **32** | 0.524330 | 0.475490 | 0.000181 |
| **33** | 0.193390 | 0.162116 | 0.644494 |
| **34** | 0.162424 | 0.322350 | 0.515226 |
| **35** | 0.524330 | 0.475490 | 0.000181 |
| **38** | 0.162424 | 0.322350 | 0.515226 |
| **41** | 0.524330 | 0.475490 | 0.000181 |
| **42** | 0.140464 | 0.827702 | 0.031833 |
| **43** | 0.162424 | 0.322350 | 0.515226 |
| **44** | 0.161148 | 0.741928 | 0.096924 |
| **46** | 0.161148 | 0.741928 | 0.096924 |
| **51** | 0.511549 | 0.004120 | 0.484330 |
| **53** | 0.940407 | 0.059275 | 0.000318 |
| **54** | 0.940407 | 0.059275 | 0.000318 |
| **58** | 0.511549 | 0.004120 | 0.484330 |
| **60** | 0.940407 | 0.059275 | 0.000318 |
| **61** | 0.193390 | 0.162116 | 0.644494 |
| **62** | 0.161148 | 0.741928 | 0.096924 |
| **63** | 0.524330 | 0.475490 | 0.000181 |
| **65** | 0.161148 | 0.741928 | 0.096924 |
| **66** | 0.002223 | 0.565120 | 0.432657 |
| **74** | 0.001355 | 0.001454 | 0.997191 |
| **75** | 0.002223 | 0.565120 | 0.432657 |
| **76** | 0.193390 | 0.162116 | 0.644494 |
| **77** | 0.524330 | 0.475490 | 0.000181 |
| **78** | 0.209646 | 0.000001 | 0.790353 |
| **79** | 0.511549 | 0.004120 | 0.484330 |
| **83** | 0.162424 | 0.322350 | 0.515226 |
| **86** | 0.161148 | 0.741928 | 0.096924 |
| **87** | 0.940407 | 0.059275 | 0.000318 |
| **88** | 0.524330 | 0.475490 | 0.000181 |
| **89** | 0.524330 | 0.475490 | 0.000181 |
| **92** | 0.193390 | 0.162116 | 0.644494 |
| **97** | 0.161148 | 0.741928 | 0.096924 |
| **102** | 0.940407 | 0.059275 | 0.000318 |
| **106** | 0.524330 | 0.475490 | 0.000181 |
| **109** | 0.524330 | 0.475490 | 0.000181 |
| **110** | 0.161148 | 0.741928 | 0.096924 |
| **111** | 0.524330 | 0.475490 | 0.000181 |
| **112** | 0.637534 | 0.128079 | 0.234387 |
| **113** | 0.637534 | 0.128079 | 0.234387 |
| **114** | 0.524330 | 0.475490 | 0.000181 |
| **117** | 0.524330 | 0.475490 | 0.000181 |
| **118** | 0.524330 | 0.475490 | 0.000181 |
| **119** | 0.193390 | 0.162116 | 0.644494 |
| **125** | 0.524330 | 0.475490 | 0.000181 |
| **127** | 0.524330 | 0.475490 | 0.000181 |
| **128** | 0.524330 | 0.475490 | 0.000181 |
| **129** | 0.548664 | 0.451152 | 0.000184 |
| **130** | 0.002223 | 0.565120 | 0.432657 |
| **135** | 0.002223 | 0.565120 | 0.432657 |
| **136** | 0.524330 | 0.475490 | 0.000181 |
| **137** | 0.524330 | 0.475490 | 0.000181 |
| **138** | 0.161148 | 0.741928 | 0.096924 |
| **139** | 0.161148 | 0.741928 | 0.096924 |
| **140** | 0.161148 | 0.741928 | 0.096924 |
| **141** | 0.644265 | 0.029003 | 0.326732 |
| **142** | 0.940407 | 0.059275 | 0.000318 |
| **144** | 0.524330 | 0.475490 | 0.000181 |
| **145** | 0.173401 | 0.462347 | 0.364252 |
| **146** | 0.193390 | 0.162116 | 0.644494 |
| **147** | 0.440517 | 0.558881 | 0.000602 |
| **148** | 0.524330 | 0.475490 | 0.000181 |
| **151** | 0.524330 | 0.475490 | 0.000181 |
| **158** | 0.524330 | 0.475490 | 0.000181 |
| **161** | 0.524330 | 0.475490 | 0.000181 |
| **163** | 0.940407 | 0.059275 | 0.000318 |
| **169** | 0.524330 | 0.475490 | 0.000181 |
| **170** | 0.524330 | 0.475490 | 0.000181 |
| **171** | 0.524330 | 0.475490 | 0.000181 |
| **172** | 0.193390 | 0.162116 | 0.644494 |
| **173** | 0.193390 | 0.162116 | 0.644494 |
| **176** | 0.524330 | 0.475490 | 0.000181 |
| **177** | 0.524330 | 0.475490 | 0.000181 |
| **179** | 0.524330 | 0.475490 | 0.000181 |
| **181** | 0.524330 | 0.475490 | 0.000181 |
| **182** | 0.024721 | 0.308502 | 0.666777 |
| **183** | 0.954527 | 0.025892 | 0.019580 |
| **186** | 0.524330 | 0.475490 | 0.000181 |
| **189** | 0.524330 | 0.475490 | 0.000181 |
| **190** | 0.524330 | 0.475490 | 0.000181 |
| **193** | 0.548664 | 0.451152 | 0.000184 |
| **195** | 0.002223 | 0.565120 | 0.432657 |
